# Acute Effects of Nicotine on Physiological Responses and Sport Performance in Healthy Baseball Players

**DOI:** 10.3390/ijerph19010515

**Published:** 2022-01-04

**Authors:** Shih-Hua Fang, Chi-Cheng Lu, Hua-Wei Lin, Kuan-Chen Kuo, Chen-Yu Sun, Yi-Ying Chen, Wen-Dien Chang

**Affiliations:** 1 Department of Sport Performance, National Taiwan University of Sport, Taichung 404401, Taiwan; shfang@ntus.edu.tw (S.-H.F.); a722353@ntus.edu.tw (C.-C.L.); e1451212@gmail.com (K.-C.K.); david91523@gmail.com (C.-Y.S.); c252522002@gmail.com (Y.-Y.C.); 2 Department of Ball Sport, National Taiwan University of Sport, Taichung 404401, Taiwan; hwlin@ntus.edu.tw

**Keywords:** nicotine, heart rate variability, cognitive function, baseball-hitting performance

## Abstract

There is interest in whether nicotine could enhance attention in sporting performance, but evidence on the acute effect of nicotine on physical response and sports performance in baseball players remains scant. This was an observational study to examine whether nicotine gum chewed before exercise could provide acute effects on physiological responses and sport performance. Accordingly, heart rate variability (HRV), saliva cotinine concentration and α-amylase activity, cognitive function, muscle strength, and baseball-hitting performance were measured. Thirteen healthy male non-smoker baseball players were recruited. Conducting two sequences with 7-day intervals, they chewed nicotine gum (nicotine group) or flavor-matched placebo gum (placebo group) for 30 min. HRV and saliva analyses were conducted before gum consumption (S1), after gum consumption (S2), and after test completion (S3). Cognitive, muscle strength, and baseball-hitting performance tests were performed after nicotine or placebo gum chewing. The outcomes of all assessed variables were compared within and between the groups. Significant changes in HRV, α-amylase, testosterone, and cortisol were observed in the nicotine group at S2 and S3 (*p* < 0.05). Compared with the placebo group, the nicotine group exhibited enhanced motor reaction times, grooved pegboard test (GPT) results on cognitive function, and baseball-hitting performance, and small effect sizes were noted (d = 0.47, 0.46 and 0.41, respectively). Nicotine could induce changes in endocrine and sympathetic nerve activity and enhance cognitive function and baseball-hitting performance. However, no increase in muscle strength was observed after nicotine intake.

## 1. Introduction

Nicotine acetylcholine receptors (nAChRs) are distributed throughout the central nervous system as well as in peripheral tissues. Activation of nAChRs enhances sensory–cognitive function in humans and animals, but the corresponding neural mechanisms are not fully understood [1]. Nicotine activates the hypothalamic–pituitary–adrenocortical (HPA) axis and increases corticotrophin-releasing hormone, arginine vasopressin, beta-endorphin, and cortisol levels [2]. In addition, nicotine activates the sympathetic–adrenal–medullary system, which may induce changes in heart rate (HR), blood pressure, and salivary α-amylase activity; the secretion of α-amylase is under strong neurohormonal control [3]. Nicotine decreases heart rate variability (HRV) by acting upon the autonomic nervous system [4].

Sporting events, especially baseball events, have long been associated with tobacco due to the considerable sponsorship funding provided by tobacco companies. However, divergent views abound as to whether tobacco can improve concentration; this is because evidence of tobacco’s relationship with sports performance is insufficient [5]. Although nicotine is not considered a doping agent, the controversial effect of smokeless tobacco and nicotine on exercise performance in elite athletes has drawn the attention of researchers in the sports field [6]. Studies have reported that tobacco-induced changes in blood distribution throughout the body may help supply adequate oxygen to skeletal muscles [6,7], engendering a considerable improvement in endurance performance. In addition, considerable enhancements in force production, and peak and average power output of lower extremities have been observed following nicotine administration [8,9]. However, studies have observed that snus is an oral-consumed and smokeless traditional tobacco, and its use does not affect muscle contractions, fatigue perception, or time to exhaustion [7,10]. Pulmonary function was lower in active smokers than in non-smokers in skill and power sports [11]. Although Guo et al. has indicated that the secretion and production of testosterone might be regulated by the hypothalamic–pituitary–gonadal (HPG) axis through the neuroendocrine system [12], other report of Peak et al. revealed that testosterone had no direct relationship with HPG axis-related hormones [13]. Therefore, the effects of nicotine on muscle strength, psychophysiological responses, and sports performance are still unproven.

Evidence indicates that chewing gum can enhance attention, in addition to promoting work performance [14]. The relationship between chewing and cognitive function was investigated through electroencephalography (EEG), and the results revealed that alpha power was increased after chewing [15]. The major concerns about smokeless tobacco are cancer risks and oral pathology [16]. However, the custom of tobacco chewing in baseball can be traced back to many years ago, when baseball players began chewing tobacco to avoid developing excessively dry mouths in dusty playing areas [17,18]. The players would chew tobacco and consume nicotine simultaneously. Previous studies supported that consumption of nicotine in the form of tobacco chewing or nicotine gum is considered to benefit sports practice and performance [6,7,14]. Nicotine gum is a relatively safer sports supplementation than tobacco chewing for health conditions [14]. Accordingly, the purpose of the present study was to assess whether nicotine gum use during exercise provides acute effects in physiological responses and sport performances. We hypothesized that nicotine would enhance the HRV, saliva cotinine concentration and α-amylase activity, cognitive function, muscle strength, and baseball-hitting performance of baseball players.

## 2. Materials and Methods

### 2.1. Participants

This was an observational study. Thirteen healthy males who had never smoked were recruited through word of mouth from the baseball team of the National Taiwan University of Sport. The participants were required to meet the following criteria: (1) be baseball players; (2) possess national-level sport performance; and (3) have continually trained for ≥3 h, four times per week for the previous ≥9 years. Participants were excluded if they had cardiac disease histories, were injured, or were unable to participate in normal training (*n* = 0). Therefore, the qualified baseball players were all included in this study (*n* = 13). A previous study suggested that a sample size of 6 would yield a power of 0.8 (with two-tailed alpha of 0.05), which is sufficient to detect effects of nicotine on HRV [8]. Accordingly, the sample of 13 participants in the present study was sufficient. We planned to recruit the baseball players as participants, and all of them were advised to abstain from stimulants such as coffee or energy drinks for the 12 h prior to the trial. Each participant was fully informed of all potential risks and experimental procedures, after which informed written consent was obtained. All experimental procedures and protocols were approved by the Institutional Human Ethics Committee in a hospital.

### 2.2. Experimental Protocol and Measures

**Figure 1:**
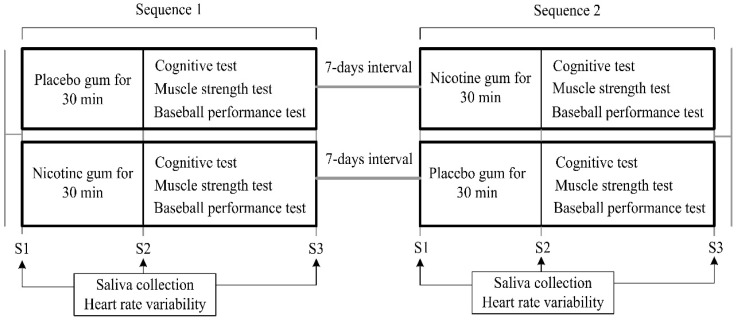
Experimental protocol.

The experimental protocol (Figure 1) was a crossover design that was implemented during 7 days. On arrival at the laboratory, each of the participants was fitted with an HR monitor (Polar V800, Kempele, Finland) and seated for 5 min, after which a baseline HR measurement was taken separately at stages 1–3. Initially, the participants were requested to remain in a seated position, and saliva samples were collected (Stage 1, S1). For the experimental trials, the participants chewed 2 mg nicotine gum (Nicotine group) or flavor-matched placebo gum (Placebo group) for 30 min while seated, and the participants’ saliva was then collected again (Stage 2, S2). Low-dose nicotine (2 mg) was proved to raise the levels of physiological and central nervous system activity [19]. Therefore, 2 mg nicotine gum was used in the current study. They then completed the Cognitrone (COG) test and the grooved pegboard test (GPT) from the Vienna Test System (VTS) as well as a muscle strength test. Furthermore, their baseball-hitting performances with respect to concentration and speed were measured. When the participants had completed all the tests, their saliva samples were collected (Stage 3, S3). All experimental trials were conducted at the same time of day; the participants were instructed to abstain from alcohol or any exercise and to only pursue habitual caffeine use (because abstinence would in itself confound withdrawal effects) on the day of and prior to any experimental trial. Additionally, to ensure similar metabolic states, the participants were asked to replicate the diet they consumed immediately prior to the first experimental visit in the lead-up to subsequent trials.

### 2.3. Nicotine Intervention

The participants were instructed to chew the gum for 30 min, according to the manufacturer’s recommendations. One piece of 2 mg mint-flavored nicotine gum or placebo gum (Nicorette Icy Mint, Johnson and Johnson Pacific, Auckland, New Zealand) was provided to the participants, followed by the following instructions: “chew until there is a strong taste, then place between your cheek and gums, and chew again when the taste has faded.”

### 2.4. Assessments

#### 2.4.1. Heart Rate Variability Measurements

Beat-to-beat HR was recorded using a portable HR monitor with a 1 ms resolution (Polar V800, Kempele, Finland). HRV-related variables, including HR, percentage of differences between adjacent normal RR intervals of >50 ms (pNN50), normalized units of low frequency (LF), and normalized units of high frequency (HF) were calculated from 5-min recordings. LF/HF was calculated from the ratio of LF to HF. Spectral analysis was performed through the maximum entropy method, and autoregressive coefficients were estimated using the Burg algorithm. Power spectra were derived over the frequency range of 0.01–0.40 Hz at a 0.01 Hz frequency resolution. LF and HF components were calculated through the integration of power spectra over the frequency range of 0.04–0.15 Hz and those over the frequency range of 0.15–0.40 Hz, respectively [4].

#### 2.4.2. Saliva Collection and Assay

Unstimulated whole saliva was collected as described previously [20]. All participants were seated and asked to thoroughly rinse their mouths with 30 mL of sterile distilled water before sample collection. The participants remained seated for 10 min, and then saliva samples (2 mL) were collected separately at three time points: before chewing the gum (S1), after chewing the gum (S2), and after all the tests were completed (S3). The saliva samples were stored immediately at −80 °C until assay. The half-life of nicotine is short; therefore, cotinine, a major metabolite of nicotine, was used as a long half-life and reliable marker. The salivary cotinine level was determined using an enzyme-linked immunosorbent assay (ELISA) kit (Cozart Bioscience Ltd., Oxfordshire, UK). Moreover, α-amylase activity and the concentrations of cortisol and testosterone were measured as described previously [20]. Specifically, α-amylase activity was determined using a kinetic reaction assay kit (Salimetrics LLC, State College, PA, USA) according to the manufacturer’s instruction. Commercial ELISA kits (DRG Instruments, GmbH, Marburg, Germany) were used to measure cortisol and testosterone concentrations. All samples were measured in triplicate, and data were expressed as absolute concentrations. The intra-assay coefficients of variation for the measurements of cotinine, α-amylase activity, cortisol, and testosterone were 5%, 4%, 4%, and 4%, respectively.

#### 2.4.3. Cognitive Function Assessments

The cognitive function was assessed using the COG test and GPT from the VTS [21]. In the COG test, attention and concentration are measured by comparing the congruency of various figures on a computer screen. In this study, the participants were presented with an abstract figure and were asked to match the figure to a model. The mean time at which “correct rejection” was achieved within a total working time of 7 min was recorded. The GPT is a test of manual dexterity, processing speed, and hand–eye coordination. The GPT involves 25 holes with randomly positioned slots. In the trial, the participants were asked, using only one hand, to put the pegs in the board in a fixed order and in the correct direction. They were encouraged to perform the task as quickly as possible. The total time was recorded in seconds. Each participant was tested twice, once with his dominant hand and once with his non-dominant hand. Subsequently, the average time was calculated to derive an overall score.

#### 2.4.4. Muscle Strength Test

A MicroFET-3 dynamometer (Hoggin Health Industries, Draper, UT, USA) was used to record the maximal voluntary isometric contraction strength of five muscles: the deltoid, biceps brachii, triceps brachii, wrist flexor, and wrist extensor muscles. For the deltoid, biceps brachii, wrist flexor, and wrist extensor muscles, the participants were seated for the measurement of the isometric force of their shoulders in 90° abduction, elbow in 90° flexion, wrist flexion in forearm pronation, and wrist extension in forearm supination, respectively. The measurement for the triceps brachii muscle was performed in the prone position, and the tested forearm was hung over the side of the treatment table for elbow extensor isometric contraction testing. The tests were repeated twice on all three occasions with 1 min rest intervals between the tests, and the bilateral tested muscles were recorded. Absolute strength and relative strength, which were calculated as absolute strength relative to body weight, were analyzed.

#### 2.4.5. Baseball-Hitting Performance Test

The participants each used a single testing bat, owned by themselves, for all the tests. Before the experiment, they all completed adequate warm-up. For the concentration test, two batting tees separated by 85.09 cm were arranged in a straight line. The heights of the batting tees were adjusted according to the position of the participant’s best hitting point. The front and back batting tees were adjusted to the same height. A baseball was placed on top of each tee, and the participant was required to swing to hit the baseball on the back tee, strike the ball forward and subsequently knock loose the baseball on the front batting tee. As long as the ball from the back tee touched the ball on the front one, the attempt was considered successful. If the ball merely knocked the front batting tee itself or other factors caused the baseball on the front tee to fall, the attempt was considered a failure. Concentration performance was measured by counting the average percentage of hits over 10 attempts over 2 separate days. For the speed test, each participant was required to swing at a total of 10 balls and to swing at each ball within 12 s of striking the last one. We used a Blast Motion baseball swing sensor (Blast Motion Inc., Carlsbad, CA, USA) that was mounted on the tail of a bat, and the data recorded during the swing were wirelessly transmitted to our mobile devices via Bluetooth. The tee mode in the swing sensor was used in collecting each participant’s swing speed data. The same test was performed on two separate days during the week-long study period to obtain the average hitting performance assessments for each group.

### 2.5. Statistical Analyses

The demographic data and all assessed variables are presented as the mean ± standard deviation. A paired *t*-test was used to determine the differences in demographic data between the nicotine and placebo gum groups. A comparative analysis of the outcome variables of saliva collection and heart rate variability were conducted using two-way repeated measure analysis of variance (condition × group), followed by the Bonferroni post hoc test. The between-group factor was group, with two groups (nicotine and placebo groups), and the within-group factor was time, with three conditions (S1, S2, and S3). For the outcome variables of cognitive function assessments, muscle strength test, baseball-hitting performance test, a paired *t*-test was conducted to compare between two groups. According to the scale presented by Cohen et al. [22], effect sizes (d) for the comparison of nicotine and non-nicotine use in terms of cognitive function, muscle strength, and baseball-hitting performance were classified into <0.2 (very small), 0.2–0.5 (small), 0.5–0.8 (medium), and >0.8 (large). The effect sizes (*p*η^2^) in terms of HRV and saliva analysis were evaluated as 0.01–0.06 (small), 0.06–0.14 (medium), and >0.14 (large) [22]. The relationship between the groups in terms of changes in baseball swing speed over the 10 baseball hit attempts was analyzed using simple linear regression. The significance level was set to α = 0.05 for all tests. All statistical analyses were performed using SPSS software (version 25; SPSS Inc., Chicago, IL, USA).

## 3. Results

**Figure 2:**
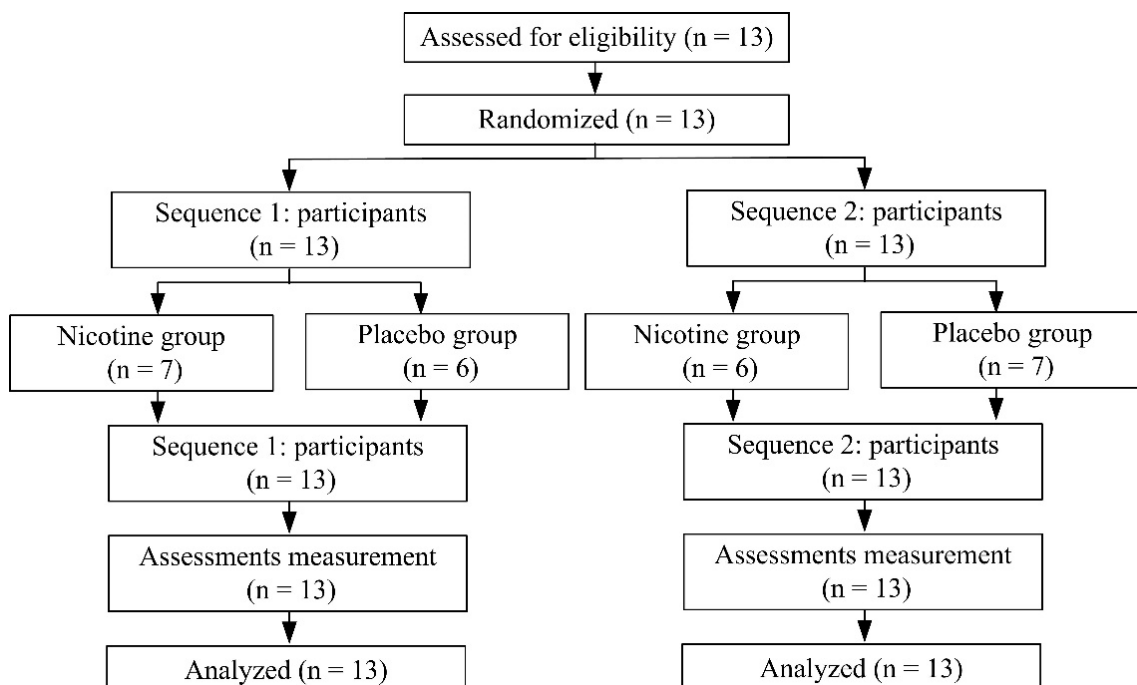
Participants disposition.

Thirteen participants were recruited in this study, and they were randomly assigned to one of two groups throughout the study. All participants completed the study without reporting negative side effects (Figure 2). The participants’ demographics are summarized in Table 1. The mean salivary cotinine concentration at 30 min after nicotine supplement was 11.2 ± 1.5 ng/mL and was undetectable at S1 and S3.

### 3.1. Effects of Nicotine and Exercise on Heart Rate Variability (HRV) and Saliva α-Amylase Activity

The changes in HRV-related variables in both groups at S1, S2, and S3 are presented in Table 2. For HR, we noted the main interaction effects of condition × group (F = 3.94, *p* = 0.04), group (F = 0.91, *p* = 0.35), and condition (F = 59.37, *p* = 0.001). For LF, HF, and LF/HF, we also discovered main interaction effects of condition × group (F = 0.37, *p* = 0.69; F = 0.37, *p* = 0.68; F = 0.49, *p* = 0.54, respectively), group (F = 0.08, *p* = 0.77; F = 0.08, *p* = 0.76; F = 0.001, *p* = 0.98, respectively), and condition (F = 7.04, *p* = 0.002; F = 7.17, *p* = 0.006; F = 8.50, *p* = 0.006, respectively). Post-hoc tests revealed that HR and LF/HF at S2 were significantly higher than those at S1 in the nicotine group. This trend was maintained until the participants completed the baseball-hitting test. Moreover, although LF increased, both pNN50 and HF decreased significantly at S3 in the nicotine group. By contrast, HR and LF/HF were significantly higher than their baseline levels only after the sports performance test (at S3) in the placebo group. In addition, the main interaction effects of condition × group (F = 5.26, *p* = 0.009), group (F = 3.18, *p* = 0.08), and condition (F = 3.16, *p* = 0.05) were noted for saliva α-amylase activity. Post hoc tests indicated that saliva α-amylase activity in the nicotine group was significantly increased at S2 (158.03 ± 85.58 vs. 129.59 ± 69.17 U/mL, *p* = 0.029, Table 2) and S3 (170.62 ± 99.11 vs. 129.59 ± 69.17 U/mL, *p* < 0.01) relative to the baseline (S1) level. Additionally, the nicotine group exhibited significantly higher α-amylase activity levels than did the placebo group at S2 and S3 (*p* = 0.02 and *p* = 0.02, respectively). In the placebo group, no difference in α-amylase activity was observed between the three time points.

### 3.2. Effects of Nicotine and Exercise on Salivary Testosterone and Cortisol

Table 2 also presents changes in salivary testosterone and cortisol in both groups at S1, S2, and S3. Baseline testosterone, cortisol, and T/C ratio measurements did not differ significantly between the nicotine and placebo groups. Regarding the testosterone and cortisol concentrations, we noted main interaction effects of condition × group (F = 2.40, *p* = 0.10; F = 3.89, *p* = 0.02, respectively), group (F = 2.90, *p* = 0.10; F = 0.81, *p* = 0.37, respectively), and condition (F = 2.42, *p* = 0.11; F = 12.28, *p* = 0.001, respectively). For the T/C ratio, main interaction effects of condition × group (F = 4.69, *p* = 0.02), group (F = 0.05, *p* = 0.83), and condition (F = 8.77, *p* = 0.004) were observed. The testosterone concentration at S2 was significantly lower than that at S1 in the nicotine group. Furthermore, the cortisol concentration at S2 was significantly lower than that at S1 in the placebo group. In both groups, the cortisol concentrations at S3 were significantly lower than the baseline concentrations. The T/C ratio at S2 was significantly lower than that at S1 in the nicotine group. In both groups, the T/C ratios at S3 were significantly higher than those at S1, but no significant difference was observed between the two groups.

### 3.3. Effect of Nicotine on Cognitive Performance

After chewing nicotine gum for 30 min, the participants performed the cognitive function tests. The mean motor reaction time of the nicotine group was significantly shorter than that of the placebo group (134.80 ± 33.78 vs. 151.70 ± 38.04 ms, d = −0.47, 95% CI = −1.24 to 0.31, *p* = 0.03; Table 3). Moreover, the nicotine group completed the GPT significantly faster than the placebo group did (121.30 ± 15.03 vs. 128.66 ± 15.81 s, d = −0.46, 95% CI = −1.25 to 0.30, *p* = 0.04).

### 3.4. Effect of Nicotine on Muscle Strength

After the participants chewed the gum, whether nicotine or placebo for 30 min, the absolute and relative muscle strength levels were similar in the left and right deltoid, biceps brachii, triceps brachii, wrist flexor, and wrist extensor muscles (all *p* > 0.05; Table 4).

### 3.5. Effect of Nicotine on Baseball-Hitting Performance

**Figure 3:**
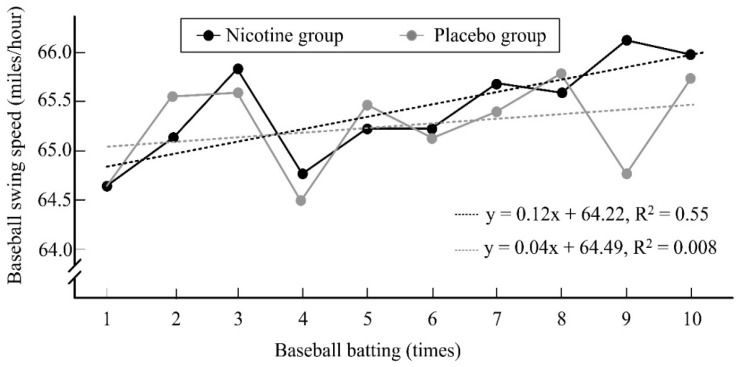
The changes over baseball batting time on the slope of baseball swing speed in both groups.

The hit percentage among the participants in the nicotine group was significantly higher than that among those in the placebo group (33.00% ± 23.71% vs. 24.50% ± 17.71%, d = 0.41, 95% CI = −0.37 to 1.18, *p* = 0.04). Furthermore, the changes in the slopes of baseball bat swing speed in the nicotine group (R^2^ = 0.55) and placebo groups (R^2^ = 0.008) increased over the 10 hit attempts, indicating a higher bat swing speed with increasing hitting time in both groups (Figure 3). However, compared with that in the placebo group, the slope in the nicotine group had a higher decreasing trend, indicating a higher increase in bat swing speed with increasing hitting time.

## 4. Discussion

We determined the effects of nicotine on cognitive function, muscle strength, and baseball-hitting performance enhancement. To the best of our knowledge, this is the first crossover and controlled trial to verify the effects of nicotine gum supplementation in baseball players. The results indicate significant changes in HRV, α-amylase activity, testosterone concentration, and cortisol concentration in the nicotine group. Compared with the placebo group, the nicotine group exhibited significantly enhanced cognitive function and baseball-hitting performance. Small effect sizes for cognitive function and baseball-hitting performance were demonstrated. However, an increase in muscle strength was not observed after nicotine intake.

We also noted that HR significantly increased in the participants after 30 min nicotine gum chewing. The low-dose nicotine could cause tachycardia by acutely stimulating the sinoatrial nodes [23], and our results support this finding. Fernandez et al. indicated that nicotine could cause sympathetic effects that induce the acute response of HR increase [24]. Our HRV analysis revealed higher HR and LF/HF measurements after exposure to nicotine, a finding that is consistent with that reported by Druyan et al. [25]. Nicotine is a potent agonist of α7 receptors, which are associated with vagal function, and generally causes sympathetic nerve overactivity [26]. Hence, our study revealed a significant increase in LF and a decrease in HF at S3. The study demonstrated sympathetic nerve activation but an inhibition of parasympathetic nerve activation. The LF/HF ratio was also increased, signifying the imbalance of sympathetic and parasympathetic nerve activity. Our findings provide support for the notion that nicotine could affect autonomic nerve activity. Moreover, α-amylase activity is a biomarker of sympathetic nerve activity. Our results reveal that α-amylase activity was significantly increased at S2 and S3, strongly endorsing the effect of nicotine use on sympathetic nerve activity. A previous review determined that the trend of salivary α-amylase activity mostly depends on the intensity of exercise [27]. In this study, no change in the participants’ α-amylase activity was observed without nicotine administration, probably because the tests of hit performance may have represented only minor exercise loading for professional baseball players.

Salivary cortisol concentration was used as an endocrine marker, and α-amylase activity was used as a sympathetic nervous system (SNS) stress marker [28]. In addition, salivary cortisol concentration and α-amylase activity are not influenced by saliva flow [29,30]. Cohen et al. demonstrated that chewing gum reduces the salivary cortisol concentration [31]. A previous study also showed that continuous chewing for more than 10 min is effective in reducing stress-induced cortisol concentrations [28], which is consistent with our finding that the cortisol concentration in the placebo group was significantly lower than that at baseline. Therefore, the non-significant change in cortisol within the nicotine group was possibly due to SNS activation caused by nicotine uptake and stress reduction caused by chewing. No significant difference in α-amylase activity was observed in the group that was administered chewing gum without nicotine [28]. According to a cross-sectional study, salivary testosterone was unrelated to salivary cotinine concentration [32]. However, other studies have found a positive [33,34] or negative [35] correlation between testosterone and nicotine exposure. In this study, we observed that the salivary testosterone concentration decreased after nicotine gum chewing and returned to the baseline concentration after exercise. The T/C ratio decreased significantly due to the greater decrease in testosterone and lower decrease in cortisol. The T/C ratio reflects the interaction between the HPA and HPG axes [36], which might be affected by nicotine use and exercise intensity. This mechanism is unclear and requires further investigation.

In clinical medicinal practice, nicotine is used to treat nicotine dependence in smokers. Additionally, a study demonstrated that the use of nicotine gum, patches, and nasal sprays in replacement therapy resulted in the improvement of successful smoking cessation [37]. Nicotine also significantly improved the signal detection of never-smokers in a visuospatial attention task [38]. A meta-analysis study determined nicotine to have significant positive effects on six domains: fine motor control, alerting attention, orienting attention, short-term episodic memory, and working memory [39]. Furthermore, nicotine was reported to relieve certain attentional and cognitive deficits associated with Alzheimer’s disease, clinical symptoms of attention-deficient hyperactivity disorder, and clinical symptoms of Parkinson’s disease [40]. A systemic review demonstrated positive evidence on the effects of nicotine on attention and fine motor control in smokers, and the corresponding meta-analysis revealed a relatively small effect size (d = 0.13–0.34) for alerting and orienting attention in the smokers compared with healthy non-smokers [39]. Our study also revealed increases in fine motor accuracy and reaction times and orienting attention in the nicotine group. Specifically, our result show that the reaction time and GPT time in the nicotine group were significantly shorter than those in the placebo group (*p* < 0.05) and demonstrated small effect sizes (d = −0.47 and −0.46, respectively). Newhouse et al. also argued that nicotine could improve attention and cognitive performance and that it could be used to manage psychiatric and neurological conditions [41]. Furthermore, our findings indicate that the nicotine group had higher cognitive performance than the placebo group did. We observed that the hit percentage of the nicotine group was significantly higher than that of the placebo group. The mechanism of attention involves a top-down process of cognitive control and bottom-up process of stimulus processing under cross-connected neurotransmitters. Moreover, nAChRs are involved in agonistic actions, and the α7 receptors of nicotine play a notable role in the context of attention [42]. The acute effect of nicotine on cognitive performance was attributed to the fact that nicotine could stimulate acetylcholine receptors and increase bottom-up stimulus processing [43].

Escher et al. compared the muscle strength of athletes who were habitual users of and those who had never used smokeless tobacco, and discovered that the maximal force and rate of force development were decreased by 12% and 9–10%, respectively, in the smokeless tobacco users [44]. Siafaka et al. indicated that a decrease in vascular reactivity and a decrease in peripheral vasoconstriction caused declined muscular O_2_ uptake in smokers [45]. A combination of nicotine and muscarinic receptors could induce biphasic responses to elicit vasoconstriction [45]. These responses could affect muscle strength and performance. However, a previous study indicated no difference in leg muscle electromyography activity during maximal voluntary contractions after snus (8 mg nicotine) use in nicotine-naïve participants [10]. Our results also indicate no effect of 2-mg nicotine on absolute and relative muscle strength. A possible explanation for this finding is that the low nicotine dose could not affect maximal voluntary isometric contraction strength. Although nicotine could cause vasoconstriction to affect muscular O_2_ uptake, the intensity of the muscle strength test was not sufficient to cause an oxygen delivery problem.

Some studies have supported the notion that nicotine could alter sports performances [8,46]. However, other studies have failed to prove an improvement in sports performances after nicotine intake [44,47]. Evidence of the effect of nicotine on an increase in sporting performances is still conflicting and results are inconclusive. Our results reveal that the baseball bat swing speed in the nicotine group was significantly improved, with the corresponding effect size being small (d = 0.41). The nicotine group also had a higher increase in bat swing speed over the baseball batting time compared with the placebo group. Baseball hitting is a skill involving the upper part of the body, and an increase in muscle strength and cognitive performance is crucial to enhance baseball-hitting performance in players. We noted that the participants’ muscle strength did not increase after nicotine intake, but their attention and reaction time improved. Hence, the bat swing speed and baseball-hitting performance could have been promoted by nicotine use in the current study.

Poltavski et al. indicated the dose–response relationship of nicotine, which showed low-dose nicotine could enhance physiological action, and high-dose nicotine caused depressant occurs [48]. We also noted that the low-dose nicotine (2 mg) could influence HRV, cognitive function, and sports performance. However, only one dose of administered nicotine was tested in the current study, and the test group was relatively small. Nicotine was added to the World Anti-Doping Agency (WADA) list indicating that nicotine is a nervous stimulant, and they have suggested upgrading it to the list of prohibited substances. Nicotine is a controversial substance, and high-dose nicotine use may result in salivation, nausea, and bradyarrhythmia [49]. Many studies supported that nicotine use, especially tobacco, had higher risks of cancer, and smoking-related cancers also included the lung, oral cavity, and pharynx, larynx, esophagus, liver, etc. cancer occurs [50,51]. Nicotine or tobacco use also had an addiction risk, and long-time use had a health hazard risk [52]. Therefore, long-term or large-dose nicotine use should involve caution.

In summary, our results suggest that an enhanced state of cognition may be responsible for the hitting-enhancing effects of nicotine. The results indicate that 2 mg nicotine gum may be useful for enhancing player concentration at the time of hitting. In contrast to inhaled nicotine, nicotine gum has a much slower onset of action, reaching its peak concentration approximately 0.5 h later, and it is also less addictive than cigarettes. Therefore, unlike cigarettes, nicotine gum has a low potential for abuse [53]. Despite its strength, the present study has some limitations. First, the number of included participants represented a moderate sample size, which is lack of time to examine individual differences among participants in the current study. Second, the use of a single dose of nicotine gum was investigated, and the accurate dose for causing effects could not be proved. Finally, the simple psychomotor tests and measures of hit speed and accuracy may not have represented the complex types of reactivity, alertness, and decision-making that are necessary for successful skill performance in baseball. Further research is required to evaluate the effect of nicotine on players during a baseball game.

## 5. Conclusions

Our HRV and salivary analysis revealed that nicotine could induce endocrine and sympathetic nerve activity in healthy male baseball players who had never smoked. Compared with the placebo group, the nicotine group exhibited enhanced cognitive function (an average decrease in motor reaction time of 11.14%; an average decrease in motor reaction time of 5.72%) and baseball-hitting performance (an average increase of 34.69%), and small effect sizes were observed for these results. However, muscle strength did not increase after nicotine intake.

## Figures and Tables

**Table 1:** All participants’ demographics.

	Participants (*n* = 13)
Gender (male), *n* (%)	13 (100%)
Age (years)	20.69 ± 0.75
Height (m)	174.46 ± 3.89
Weight (kg)	78.12 ± 10.34
Body mass index (kg/m^2^)	25.61 ± 2.77
Baseball age (years)	11.31 ± 1.38

**Table 2:** The heart rate variability (HRV), and the levels of testosterone, cortisol and T/C ratio at different stages of both groups.

	Nicotine Group			Placebo Group			Condition Effect		Time Effect		Condition * Time	
	S1	S2	S3	S1	S2	S3	*p*	η*p*^2^	*p*	η*p*^2^	*p*	η*p*^2^
Heart rate variability												
HR (bpm)	74.11 ± 10.88	80.77 ± 10.12 ***, #	93.95 ± 11.49 ***	76.69 ± 7.21	74.55 ± 7.56	92.49 ± 12.26 ***	0.35	0.04	0.001	0.73	0.04	0.15
pNN50 (%)	23.33 ± 22.71	17.28 ± 17.37	5.27 ± 4.03 **	17.36 ± 16.90	20.64 ± 15.12	12.54 ± 18.44	0.54	0.01	0.01	0.22	0.007	0.20
LF (n.u.)	60.74 ± 13.00	64.81 ± 16.17	73.58 ± 15.57 **	61.79 ± 12.59	65.10 ± 10.96	69.11 ± 17.86	0.77	0.004	0.002	0.24	0.69	0.01
HF (n.u.)	39.09 ± 12.88	35.04 ± 16.05	26.21 ± 15.37 **	38.03 ± 12.56	34.79 ± 10.95	30.68 ± 17.64	0.76	0.004	0.006	0.24	0.68	0.01
LF/HF	1.84 ± 0.95	2.67 ± 2.21 *	4.63 ± 3.88 **	1.97 ± 1.17	2.16 ± 1.05	4.70 ± 5.94 *	0.98	0.001	0.006	0.27	0.54	0.02
α-amylase (U/mL)	129.59 ± 69.17	158.03 ± 85.58 *, #	170.62 ± 99.11 *, #	139.33 ± 94.31	122.30 ± 78.46	143.60 ± 103.17	0.08	0.13	0.05	0.13	0.009	0.20
Testosterone(nmole/L)	0.78 ± 0.31	0.53 ± 0.30 **, #	0.79 ± 0.43	0.86 ± 0.36	0.81 ± 0.35	0.81 ± 0.40	0.10	0.11	0.11	0.09	0.10	0.09
Cortisol (nmole/L)	3.48 ± 2.10	3.54 ± 1.75 #	2.33 ± 1.22 **	3.53 ± 1.58	2.59 ± 0.99 **	2.37 ± 1.22 **	0.37	0.03	0.001	0.34	0.02	0.14
T/C ratio	0.30 ± 0.21	0.18 ± 0.13 *, ##	0.52 ± 0.50 **	0.27 ± 0.11	0.34 ± 0.17	0.43 ± 0.29 *	0.83	0.002	0.004	0.27	0.02	0.16

* *p* < 0.05, ** *p* < 0.01, *** *p* < 0.001 compared to S1 of each group. η*p*^2^ = Partial eta squared. # *p* < 0.05, ## *p* < 0.01 compared to placebo group at the same time point. Data expressed as mean ± SD. LF: low frequency; HF: high frequency; T/C: Testosterone/Cortisol.

**Table 3:** The cognitive function in both groups.

	Nicotine Group	Placebo Group	d	*p*
Reaction time				
Reaction (msec)	267.00 ± 49.89	270.70 ± 46.07	−0.07	0.53
Motor (msec)	134.80 ± 33.78	151.70 ± 38.04 #	−0.47	0.03
Cognitrone				
Correct rejection (msec)	0.70 ± 0.05	0.73 ± 0.06	−0.54	0.19
Total of correct rejections	44.20 ± 3.19	44.70 ± 3.80	−0.14	0.63
GPT (sec)	121.30 ± 15.03	128.66 ± 15.81 #	−0.47	0.04

# *p* < 0.05: compared to placebo group. GPT, groove pegboard test.

**Table 4:** Absolute and relative muscle strengths in both groups.

	Nicotine Group		Placebo Group		d ^a^	*p* ^a^	d ^b^	*p* ^b^
	Left	Right	Left	Right				
Absolute strength (Ib)								
Deltoid	62.17 ± 8.76	61.53 ± 8.61	59.20 ± 8.45	63.24 ± 7.82	0.34	0.24	−0.20	0.09
Biceps brachii	73.39 ± 13.62	68.80 ± 13.47	73.22 ± 13.60	67.39 ± 12.33	0.01	0.30	0.10	0.94
Triceps brachii	48.07 ± 7.87	50.52 ± 9.39	49.70 ± 6.20	51.98 ± 9.58	−0.23	0.47	−0.15	0.32
Wrist flexor	50.30 ± 11.81	48.03 ± 9.04	46.60 ± 9.38	49.87 ± 8.50	0.34	0.48	−0.21	0.20
Wrist extensor	67.59 ± 12.20	64.79 ± 13.83	67.11 ± 9.66	67.78 ± 10.31	0.04	0.31	−0.24	0.82
Relative strength (Ib/kg)								
Deltoid	0.80 ± 0.11	0.79 ± 0.10	0.77 ± 0.12	0.81 ± 0.09	0.26	0.23	−0.21	0.07
Biceps brachii	0.94 ± 0.12	0.88 ± 0.12	0.94 ± 0.14	0.86 ± 0.12	0.01	0.32	0.16	0.93
Triceps brachii	0.62 ± 0.11	0.65 ± 0.09	0.64 ± 0.10	0.67 ± 0.14	−0.19	0.30	−0.16	0.35
Wrist flexor	0.65 ± 0.15	0.62 ± 0.11	0.60 ± 0.13	0.65 ± 0.15	0.35	0.34	−0.22	0.22
Wrist extensor	0.87 ± 0.14	0.83 ± 0.13	0.87 ± 0.13	0.87 ± 0.12	0.01	0.24	−0.31	0.88

^a^ Nicotine vs. placebo groups in left side; ^b^ Nicotine vs. placebo groups in right side.

## Data Availability

Data is contained within the article.
